# A Quorum Sensing Regulated Small Volatile Molecule Reduces Acute Virulence and Promotes Chronic Infection Phenotypes

**DOI:** 10.1371/journal.ppat.1002192

**Published:** 2011-08-04

**Authors:** Meenu Kesarwani, Ronen Hazan, Jianxin He, YokAi Que, Yiorgos Apidianakis, Biliana Lesic, Gaoping Xiao, Valérie Dekimpe, Sylvain Milot, Eric Deziel, François Lépine, Laurence G. Rahme

**Affiliations:** 1 Department of Surgery, Harvard Medical School and Massachusetts General Hospital, Boston, Massachusetts, United States of America; 2 Shriners Burns Institute and Massachusetts General Hospital, Boston, Massachusetts, United States of America; 3 INRS-Institut Armand-Frappier, Laval, Québec, Canada; Medical College of Wisconsin, United States of America

## Abstract

A significant number of environmental microorganisms can cause serious, even fatal, acute and chronic infections in humans. The severity and outcome of each type of infection depends on the expression of specific bacterial phenotypes controlled by complex regulatory networks that sense and respond to the host environment. Although bacterial signals that contribute to a successful acute infection have been identified in a number of pathogens, the signals that mediate the onset and establishment of chronic infections have yet to be discovered. We identified a volatile, low molecular weight molecule, 2-amino acetophenone (2-AA), produced by the opportunistic human pathogen *Pseudomonas aeruginosa* that reduces bacterial virulence *in vivo* in flies and in an acute mouse infection model. 2-AA modulates the activity of the virulence regulator MvfR (**m**ultiple **v**irulence **f**actor **r**egulator) via a negative feedback loop and it promotes the emergence of *P. aeruginosa* phenotypes that likely promote chronic lung infections, including accumulation of *lasR* mutants, long-term survival at stationary phase, and persistence in a *Drosophila* infection model. We report for the first time the existence of a quorum sensing (QS) regulated volatile molecule that induces bistability phenotype by stochastically silencing acute virulence functions in *P. aeruginosa*. We propose that 2-AA mediates changes in a subpopulation of cells that facilitate the exploitation of dynamic host environments and promote gene expression changes that favor chronic infections.

## Author Summary

*P. aeruginosa* causes acute as well as chronic infections in humans. In this paper we report the identification of a *P. aeruginosa* small molecule, 2-AA, that modulates this pathogen's virulence to promote chronic infections. We show that the synthesis of 2-AA, responsible for the grape-like odor of *P. aeruginosa* cultures and of wound infections, is controlled by the **m**ultiple **v**irulence **f**actor **r**egulator (MvfR) important for virulence in acute infections. 2-AA reduces the production of MvfR-regulated acute virulence factors, and attenuates acute virulence by negatively fine-tuning the MvfR regulon activity. Moreover, we show that 2-AA adapts *P. aeruginosa* for chronic infections by promoting mutations in a key acute virulence gene (*lasR*) and by prolonging bacterial survival. The findings presented here reveal the function of a new MvfR-regulated molecule, and highlight MvfR's importance as a highly promising target for the development of inhibitors that can simultaneously halt acute and chronic infections caused by *P. aeruginosa*, and possibly by other pathogenic bacteria. This study uncovers insights that paradigmatically pave the way for the search of 2-AA-like small volatile molecules that promote pathogen adaptation and establishment of chronic infections caused by foreboding human pathogens.

## Introduction

Bacteria excrete small molecules that act as specific chemical signals to positively regulate specialized processes [1], including the production of virulence factors important for pathogenic infection, host colonization, and interspecies microbial interactions [2]. Using interconnected multi-layered regulatory networks, such as quorum sensing (QS) networks, bacteria sense and respond to external and internal bacterial cell signals as well as environmental cues, thereby adapting to exploit target hosts. Adaptation and coordination of gene expression is particularly important for pathogenic microorganisms that need to colonize changing host environments since their ability to sense and respond to host environmental cues is crucial for their survival.

*Pseudomonas aeruginosa* is an opportunistic human pathogen that causes chronic and acute infections, and is a major agent of morbidity and mortality in cystic fibrosis (CF) patients. Establishment of chronic *P. aeruginosa* respiratory or wound infections requires a complex adaptive process that mediates essential physiological changes allowing bacterial cells to survive and persist in a dynamic host environment. Although insights into chronic infection pathways have been reported [3]–[7], the specific bacterial signals that may promote the transition and/or adaptation of known pathogens from an acute to a chronic infection remain unknown.

Many *P. aeruginosa* virulence factors associated with acute infections are controlled by QS [8]. This pathogen has an extensively studied complex QS-communication network that facilitates cross-talk between organisms and impacts many *P. aeruginosa* group-related behaviors including virulence [8]. There are at least three known QS systems in *P. aeruginosa*: two are dependent on the acyl-homoserine-lactone (AHL) QS transcription factors LasR and RhlR [9], and a third is dependent on the 4-hydroxy-2-alkylquinolines (HAQs) LysR-type transcription factor MvfR (**m**ultiple **v**irulence **f**actor **r**egulator) [10]. MvfR is critical for *P. aeruginosa* acute infection through its control of genes involved in the production of secreted virulence factors and in iron assimilation [10]–[13]. This regulator controls HAQ signaling and its own activity by positively regulating the expression of genes in the *pqsABCDE* [14] and *phnAB* [10] operons. These operons encode enzymes that catalyze the biosynthesis of at least 59 distinct low molecular weight compounds, most of which are structurally related to HAQs. While two of the most abundant HAQs, [4-hydroxy-2-heptylquinoline (HHQ) and 3,4-dihydroxy-2-heptylquinoline (Pseudomonas Quinolone Signal-PQS)] [14]–[16], function *in vivo* as ligands that bind and activate MvfR [14], [17], the biological functions of other PqsABCD/PhnAB biosynthetic products remain elusive.

In this study, we show that one of these abundant MvfR-regulated non-HAQ low molecular weight molecules, 2-aminoacetophenone (2-AA), that is used to diagnose *P. aeruginosa* infections in humans [18], reduces acute virulence by negatively fine-tuning the transcription and synthesis of the MvfR ligand HHQ, and promotes changes that are critical for pathogen adaptation and important for chronic infection.

## Results/Discussion

### 2-AA Synthesis Is Controlled by MvfR but Is Not Required for the Activation of Its Regulon

**Figure 1:**
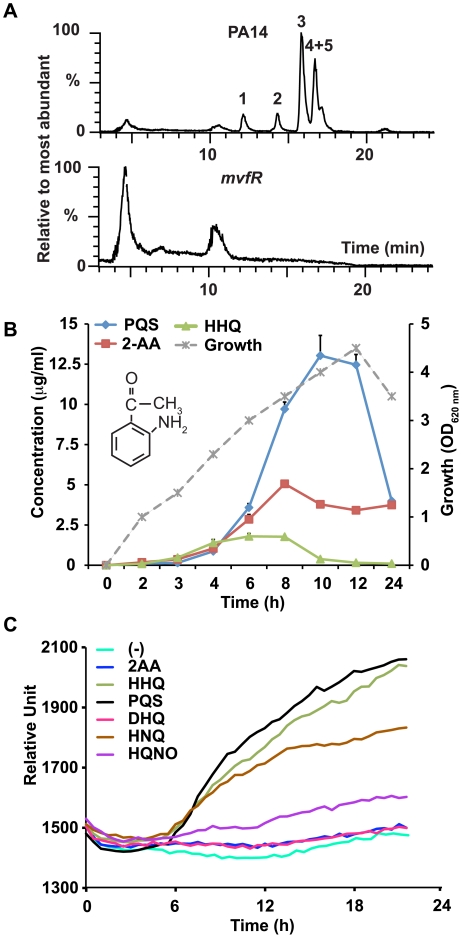
2-AA synthesis is controlled by MvfR. (A) LC/MS total ion chromatograms show the relative percentage of the most abundant small molecules in *P. aeruginosa* (PA14) (upper panel) and isogenic *mvfR* mutant (lower panel) supernatants after 9 h of growth. The most abundant molecules in PA14 cultures that were absent in *mvfR* mutant cultures were: DHQ (1); 2-AA (2); HQNO (3); and HHQ+PQS (4+5). The abundant peaks shown in *mvfR* are negligible compared to the amount of HHQ or PQS produced by PA14 since the percentage value on the Y axis is drawn with respect to the most abundant molecule detected. (B) Production kinetics of 2-AA in PA14 supernatants in LB. PA14 growth is shown as OD_600 nm_ on the secondary Y axis *versus* time. The chemical structure of 2-AA is shown in the inset. (C) 2-AA is not a MvfR co-inducer. MvfR-dependent *pqsA-*GFP(ASV) expression in the *pqsA::pqsH* mutant with or without exogenous molecules (10 µg/ml), reported as relative fluorescence *versus* time, averaged for six replicates.

To determine the functions of the abundant MvfR-regulated small molecules, we first compared the liquid chromatography/mass spectrometry (LC/MS) total ion chromatograms of culture-free supernatants from highly pathogenic wild-type *P. aeruginosa* (PA14) cells versus those of isogenic *mvfR* mutant cells. As shown in Figure 1A, the *mvfR* versus PA14 supernatant lacked HHQ and PQS, as well as three other abundant low molecular weight compounds: the HAQ molecule 4-hydroxy-2 heptylquinoline *N*-oxide (HQNO) [19], 2,4-dihydroxyquinoline (DHQ) [20], and the non-HAQ molecule 2-AA. 2-AA is a relatively simple, non-HAQ volatile molecule responsible for the grape-like odor of *P. aeruginosa* cultures as well as burn wounds infected with *P. aeruginosa* [18], [21]. Along with DHQ and HQNO, 2-AA is also produced and excreted by the HAQs-producing bacterium *Burkholderia thailandensis* [23] (Figure S1). Because HHQ and PQS both induce *pqsABCDE* expression [14], we investigated whether these three additional abundant molecules also induce expression of these genes. Maximum levels of 2-AA in the cell supernatant vary from micromolar to millimolar range depending on the growth medium used [18], [22]. We used Luria-Bertani (LB) broth media in experiments examining the effects of exogenous 2-AA supplementation on *pqs* operon gene transcription. We chose to use LB broth because it supports lower levels of 2-AA production (37.5 µM = 5 µg/ml Figure 1B) than other media [18]. Using a *pqsA-*green fluorescence protein (GFP)(ASV)-transcriptional reporter fusion in a *pqsA::H* double mutant background that does not produce any of these molecules (Figure S2), we exogenously added to LB media the above molecules. HQNO only modestly induced *pqsABCDE* expression, while DHQ and 2-AA did not induce *pqsABCDE* expression (Figure 1C). This finding indicates that unlike HHQ and PQS, 2-AA and DHQ molecules may have biological roles other than activating *pqs* operon transcription.

### 2-AA Down-regulates the Expression of MvfR-regulated Loci

**Figure 2:**
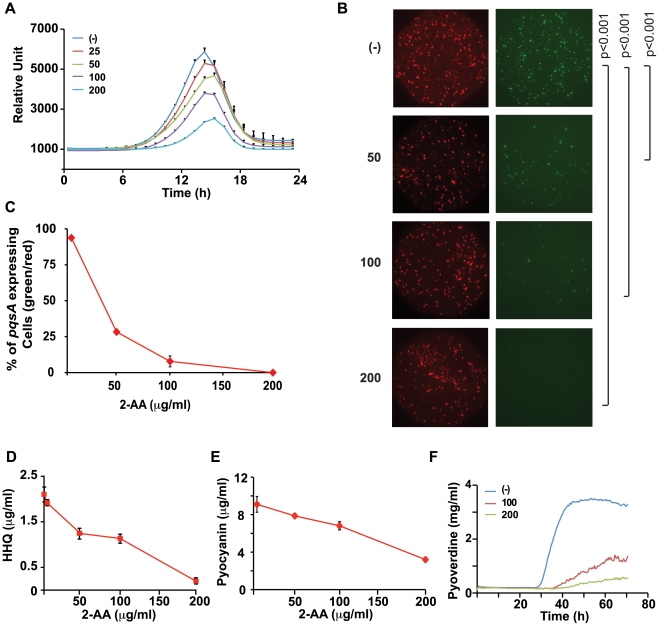
2-AA silences MvfR regulon in a subpopulation of cells and restricts HAQ mediated QS signaling relevant in acute infection. (A) Quantification of *pqsA-*GFP(ASV) expression in PA14 cells in response to increasing 2-AA concentration (µg/ml); the error bar represents the standard deviation of the 6 replicates. The difference in fluorescence intensity at the peak was statistically significant at all concentrations with *p* values <0.005. (B) Expression of *pqsA-*GFP(ASV) in response to 2-AA (µg/ml). Images of total number of cells in the optical field as assessed by membrane staining (red) and bacterial cells expressing *pqsA-*GFP(ASV)(green). (C) Quantification of *pqsA* expressing cells (percentage of green/red) in response to each 2-AA concentration. (D) Production of HHQ in supernatants collected from PA14 cells (OD 2.0) with exogenously added 2-AA at 0–200 µg/ml HHQ levels were quantified in triplicate by LC/MS. The error bars represent the standard deviation from triplicate samples. (E) Levels of pyocyanin in PA14 cultures grown (to OD 3.0) in LB and LB supplemented with varying (0–200 µg/ml) concentrations of 2-AA. Error bars represent standard deviation of the triplicate samples. These experiments were repeated at least three times with similar results. (F) Kinetics of pyoverdine production in the presence of 100 and 200 µg/ml of 2-AA. (-) indicates no exogenously added 2-AA.

The kinetics and dose-dependency effects of exogenously added 2-AA and DHQ on *pqsA* regulation were examined in greater detail using WT cells carrying the *pqsA-*GFP(ASV) transcriptional reporter fusion. Figure 2A shows that 2-AA, but not DHQ (Figure S3A), greatly reduced *pqsA* expression, in a dose-dependent manner, with 2-AA achieving the strongest inhibition at 200 µg/ml (1.5 mM). Bacterial growth was unaffected by either 2-AA or DHQ (Figure S3B and C). In light of these results, we focused our subsequent experimental efforts on 2-AA.

We visualized *pqsA*-GFP expression under a fluorescent microscope in the presence of a range of 2-AA concentrations. Surprisingly, but in accordance with the established dose-dependent effects of 2-AA, only a subpopulation of the cells showed a shut-down of *pqsA*-GFP activity in the presence of 50 µg/ml (0.375 mM) 2-AA (Figure 2B and C); a statistically significant number of cells had turned off the *pqsA* expression though some still fluoresced strongly. No fluorescing cells were seen at 200 µg/ml 2-AA (Figure 2B and C). Thus 2-AA appears to promote phenotypic heterogeneity in a genetically “homogenous” population and silence *pqs* operon expression in a fraction of the cells, suggesting that MvfR active and inactive cells co-exist to achieve a bistable phenotype [24]. The size of the 2-AA responsive population increased with higher 2-AA concentrations. The need for higher concentrations of 2-AA to produce significant detectable transcriptional changes may be due to the compound affecting only a subpopulation of cells.

2-AA inhibition of MvfR is likely mediated via negative feedback regulation given that MvfR controls 2-AA synthesis (Figure 1A). To corroborate our *pqsA* expression data, we measured levels of HHQ in 2-AA treated cells and found that HHQ production was also inhibited by 2-AA in a dose-dependent manner (Figure 2D). Consistent with the view that 2-AA down-regulates the MvfR regulon, we observed that 2-AA decreased production of pyocyanin (Figure 2E) and pyoverdine (Figure 2F), virulence factors whose synthesis depends on MvfR. A time course study of 2-AA production (Figure 1B) showed that 2-AA levels did not significantly decrease as occurs for the MvfR activators HHQ and PQS (Figure 1B), indicating that the production kinetics and stability of 2-AA are distinct from those of the MvfR activators. Together, this convergence of data strongly suggests that 2-AA has the novel biological activity of silencing the MvfR regulon.

### 2-AA Down-regulates *pqsABCDE* Operon Expression via MvfR, and by Targeting HAQ Biosynthesis Enzymes

**Figure 3:**
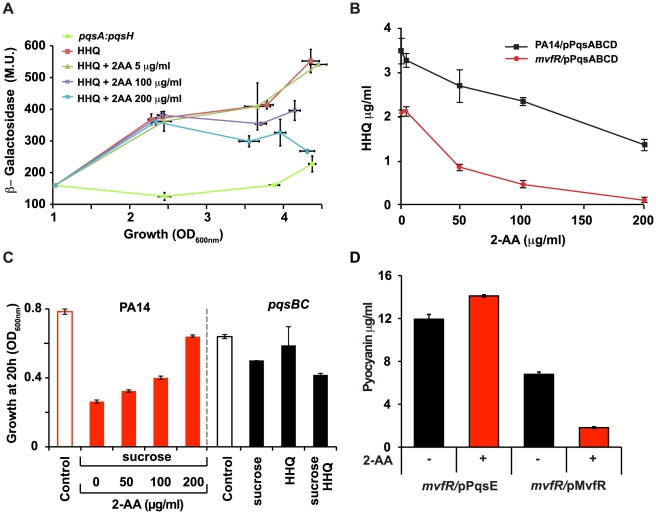
Negative regulation of the MvfR regulon by 2-AA is a result of down-regulation of *pqsABCDE* expression and interference with MvfR activity via inhibition of HHQ biosynthesis. (A) *pqsA* promoter response was assessed by measuring *pqsA*-*LacZ* expression in the PA14 isogenic *pqsA::pqsH* double mutant in the presence of HHQ (10 µg/ml) or HHQ together with 2-AA at the indicated concentrations. β-galactosidase activity is given in Miller Units, and plotted against growth assessed at OD_600 nm_. The experiment was repeated three times with similar results. (B) Levels of HHQ in the supernatant (OD 2.0) of PA14 and *mvfR* mutant cells constitutively expressing *pqsABCD* on a plasmid. HHQ levels were quantified in triplicate by LC/MS. The error bars represent the standard deviation of triplicate samples. (C) Growth of PA14 and *pqsB::C* mutant cells carrying *pqsA-SacB* after 20 h of inoculation in the presence or absence of sucrose (10%), and/or with exogenously added various concentrations of 2-AA. The *pqsA* promoter of *pqsA-SacB* in the *pqsB::C* mutant was induced by exogenous addition of 10 µg/ml HHQ where indicated. (D) Concentration of pyocyanin in *mvfR* mutant cells (OD 3.0) over-expressing MvfR or PqsE under a constitutive promoter in the presence or absence of 2-AA (200 µg/ml). Error bars represent standard deviations of triplicate samples. These experiments were repeated at least three times with similar results.

To elucidate the mechanism by which 2-AA inhibits the MvfR regulon, we administered 2-AA together with HHQ to PA14 isogenic *pqsA::pqsH* double mutant cells, which do not produce any HAQs [14] or 2-AA (Figure S2) but have functional MvfR. The expression of the *pqsA* reporter in *pqsA::pqsH* cells requires activation of MvfR. While exogenous addition of HHQ to these cells induced *pqsA* reporter expression via activation of MvfR, co-addition of 2-AA, at 100 µg/ml (0.75 mM) and above, attenuated this expression in a dose-dependent manner, suggesting that 2-AA may negatively impact the MvfR-regulated operon *pqsABCDE* at the transcriptional level (Figure 3A) via MvfR. Unlike other anthranilic acid analogs, which act on PqsA activity and inhibit the synthesis of MvfR ligands [25], the apparent 2-AA inhibition described here can be regarded as independent of PqsA since the *pqsA::pqsH* mutant was used in these experiments. Moreover, 2-AA did not perturb MvfR protein levels or MvfR ligand stability (data not shown).

To assess whether down-regulation of MvfR activity by 2-AA is due to reduced ligand levels, we engineered *mvfR* mutant cells to synthesize HHQ independently of MvfR by constitutively expressing *pqsABCD*. These cells produced HHQ in the absence of 2-AA whereas HHQ production decreased in the presence of 2-AA in a dose-dependent manner (Figure 3B), indicating that inhibition of the MvfR regulon can also occur independently of MvfR. This post-transcriptional inhibition could be mediated through interference with ligand biosynthesis.

The *pqsABBCDE* operon gene *pqsA* is required for the synthesis of 2-AA and HHQ (Figure S2). In our effort to understand 2-AA biosynthesis, we created various mutants in the PQS operon. Surprisingly, the *pqsB::C* mutant produced 2-AA in the absence of HHQ (Figure S2), demonstrating that neither *pqsB* and *pqsC* is required for 2-AA synthesis. Addition of PQS to *pqsB::C* mutant cultures induced *pqs* operon transcription, resulting in WT levels of 2-AA (Figure S2). However, as expected the addition of PQS did not result in production of HHQ (data not shown).

We used *pqsB::C* mutant cells with an additional, more sensitive reporter system to further assess the effects of endogenous 2-AA produced by the *pqsB::C* mutant on transcription of the *mvfR* regulon and its biological relevance in the absence of HHQ, as well as to quantify 2-AA effects on the *pqs* operon. We fused the *pqsA* promoter to the *Bacillus subtilis sacB* gene that codes for the levansucrase product, which is toxic when cells are grown in the presence of sucrose. The *sacB* gene has previously been incorporated into allelic exchange vectors as a means of counter-selection [23]. As shown in Figures 3C and S4, PA14 cells with the *pqsA-sacB* fusion gene incorporated stably into their chromosome did not grow in the presence of sucrose, while the corresponding isogenic *pqsB::C* mutant cells did grow significantly, corroborating the findings that 2-AA suppresses *pqsA* promoter activation and indicating that this effect is stronger in the absence of HHQ. Bacterial cell proliferation occurred even following exogenous addition of HHQ, which promotes further induction of the *pqs* operon (Figure 3C and S4). Importantly, the endogenous level of 2-AA in the *pqsB::C* mutant cells in presence of the inducer, had a suppression efficacy comparable to 100 µg/ml of exogenously added 2-AA Figure 3C. These results are physiologically important and consistent with the putative role of 2-AA in down-regulation of MvfR regulon. These results also suggest there may be limited uptake of exogenously added 2-AA thereby requiring addition of higher concentrations of 2-AA to generate a physiological response by exogenously added 2-AA.

The last gene in the *pqs* operon, *pqsE*, is essential for pyocyanin production [16], [26]–[27], but dispensable for HAQ synthesis [12], [15]. Importantly, 2-AA did not reduce pyocyanin in an *mvfR* mutant that constitutively expressed *pqsE,* even though it efficiently inhibited pyocyanin production when MvfR was constitutively expressed (Figure 3D). Hence we can deduce that 2-AA negative regulation of the MvfR regulon is a result of the down-regulation of *pqsABCDE* operon expression and interference with MvfR activity upstream of PqsE, via inhibition of HHQ biosynthesis.

### 2-AA Treatment Reduces the Mortality of PA14-infected Flies and Mice

**Figure 4:**
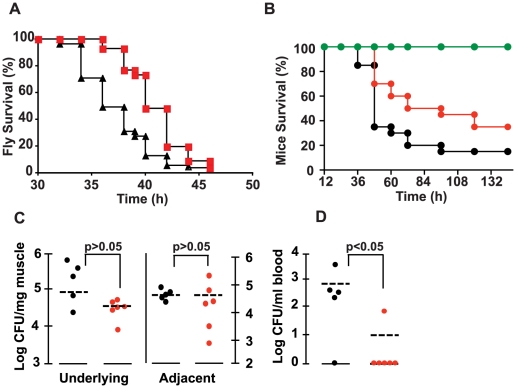
2-AA reduces *P. aeruginosa* virulence. (A) Survival kinetics of flies injected with a PA14 bacterial suspension with (∼1.6 ng/fly) and without 2-AA. Data were averaged from two experiments with n(PA14) = 55 and n(PA14+2-AA) = 56. Significance of difference of survival rate was calculated using the log-rank test of the Kaplan Meier survival estimate (*p* = 0.0056). (B) Survival rates of mice infected with PA14 *versus* PA14 + 67 µg of 2-AA/mouse. The data correspond to the averages of two independent experiments, with n(PA14) = 20 and n(PA14+2-AA) = 20. Significance of difference of survival rates was calculated using the Kaplan-Meier method (*p = *0.03), with a hazard ratio of 1.8932 (95% CI, 1.0664 to 6.0718). (C and D) Bacterial loads in the local and adjacent muscle and in blood were determined for control and 2-AA treated mice, at 20 h post-burn and infection. The statistical significance of the CFU/mg muscle tissue differences between the control and experimental mice were determined using the Mann-Whitney test for independent samples, with *p = *0.43 for the difference in the underlying and adjacent muscle in response to 2-AA, and *p* = 0.045 for the difference in the blood in response to 2-AA. CFU data are presented as log 10. Black PA14, red PA14+2-AA, green 2-AA only.

We have shown previously that *P. aeruginosa* pathogenesis can be studied in *Drosophila melanogaster* [28]–[30]. *Drosophila* shares striking similarities with mammals in terms of its overall physiology and innate immunity signal transduction pathway components [28], [31]. *P. aeruginosa* strain PA14 is highly virulent in flies, causing high mortality; meanwhile, *mvfR* mutants exhibit reduced virulence, causing reduced mortality [30]. Therefore, since 2-AA negatively regulates MvfR, we first tested whether it reduces *P. aeruginosa* virulence in *Drosophila.* As shown in the Figure 4A, flies co-injected with *P. aeruginosa* and 2-AA succumb to infection significantly later than flies injected with only *P. aeruginosa*.

To confirm the *in vivo* efficacy of 2-AA in attenuating the virulence of PA14 cells and validate the above findings in a mammalian model of infection, we used the well-studied acute mouse burn and infection model [32], in which we have shown previously that *mvfR* mutant cells cause attenuated virulence [10], [25]. Indeed, as shown in Figure 4B, 2-AA also attenuated the virulence of PA14 cells in mice. Mice inoculated with PA14 and injected once with 2-AA survived significantly longer than control mice inoculated with PA14 alone. Importantly, this effect could not have been due to reduced PA14 proliferation since 2-AA did not affect the bacterial colony forming units (CFUs) in the muscle tissue underlying the burn injury and infection site (Figure 4C). Importantly, 2-AA greatly hampered the systemic spread of bacteria in the blood of the infected mice (Figure 4D), which is a significant problem in humans with *P. aeruginosa* infections. Silencing of MvfR regulon activity, and thus of acute virulence factor gene expression, is likely responsible in large part for the reduced systemic dissemination of PA14 cells and attenuated PA14 virulence observed.

### 2-AA Promotes the Emergence of *P. aeruginosa* Phenotypes that likely Promote Chronic Lung Infections

One may wonder why a pathogen such as *P. aeruginosa* would produce a molecule that decreases its own virulence. However, given that successful adaptation of an organism depends on its ability to regulate gene expression in response to its changing environment and thereby maximize its long-term survival, the existence of such a mechanism should not be surprising. There is ongoing debate regarding the role of QS in chronic infection due to inactivation of LasR in *P. aeruginosa* isolates from CF sputum, while a lack of QS has been proposed to facilitate adaptation during a chronic infection [33]. Since 2-AA down-regulates the expression of QS-related acute virulence functions, we questioned if the same molecule could promote other functions that support adaptation during chronic infections. We therefore examined a variety of phenotypes associated with chronic infection by *P. aeruginosa*.

**Figure 5:**
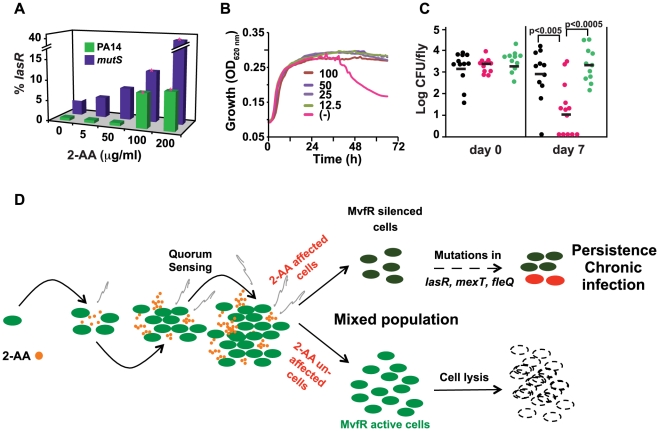
2-AA promotes phenotypes associated with pathogenic bacteria at chronic infection sites. (A) 2-AA promotes increased accumulation of *lasR* mutations in PA14 and *mutS* cells in a concentration-dependent manner. Data are averages of two independent experiments, performed in triplicate. Significance of difference in number of *lasR* mutants was calculated using Student's *t-test*, assuming equal variance. Asterisks represent significant differences relative to the untreated control group. For both PA14 and *mutS* mutant cells, *lasR* mutant accumulation was significantly increased with 100 µg/ml and 200 µg/ml 2-AA (*p's* = 0.02 and 0.0001 for PA14, and *p's = *0.05 and 0.01 for *mutS*, respectively). (B) 2-AA promotes prolonged stationary phase growth. Untreated PA14 cells began to lyse by 40 h, whereas PA14 cells treated with 2-AA at all tested concentrations did not. (C) 2-AA promotes persistent infection in flies. Bacterial CFUs per fly were assessed at 7 d post-*P. aeruginosa* feeding (6–9 flies per time point). Significant differences in CFUs after 7 d were seen for *pqsA* (pink) *versus* PA14 (black) or *pqsB::C* (green); Student's t-test *p* values are indicated in the figure. The data presented were combined from two independent experiments. (D) A model for 2-AA function. As the volatile (squiggly grey lines) 2-AA accumulates overtime in a microenvironment (i.e. biofilms, wounds or CF lungs) it creates a heterogeneous population of cells that consists of WT and physiologically and genetically different cells. The unaltered WT cells undergo lysis. On the other hand, the subpopulation of cells with silenced MvfR can continue to accumulate mutations that are beneficial for adaption to chronic infection.

A significant fraction of *P. aeruginosa* cells isolated from chronically infected CF patients accumulate multiple mutations in genes affecting acute virulence functions, including *mucA*, the regulator of alginate production, the QS regulator LasR, type III secretion system, multidrug efflux pumps, genes involved in motility, and DNA repair genes such as *mutS* [2], [34]–[38]. Mutations in the *lasR* gene are particularly notable, not only because they accumulate in bacteria colonizing the lungs of chronically infected CF patients, but also because they have been shown to promote long-term growth and survival of CF isolates *in vitro* [33]. Therefore, we first examined the frequency of occurrence of *lasR* mutations in populations of *P. aeruginosa* grown in the presence of increasing concentrations of 2-AA. We found that PA14 cells exposed to increasing 2-AA concentrations for 10 d accumulated significantly more *lasR* mutations than untreated cells (Figure 5A). Sequence analysis showed that these *lasR* mutant lines harbor simple deletions or single nucleotide non-synonymous mutations in the *lasR* gene that produce inactive LasR protein (Table 1). One of the *lasR* mutant lines also carried mutations in the intergenic region of the *fleQ* flagellar gene regulator, which is important for motility, and five clones carried mutations in the intergenic region of *mexT*, a regulator of multidrug efflux. Mutations in these genes lead to loss of flagellar motility and antibiotic resistance, respectively [39]–[40]. However, no mutations in the DNA repair gene *mutS*, in type III secretion system genes (*exsA*, *pscQ* and *popD*), or in the virulence factor *vfR* or *rpoN*, which are also associated with chronic infections [2], were identified in any of the *lasR* mutant lines sequenced. Additionally, 2-AA does not appear to be mutagenic, as it did not stimulate mutagenesis in a standard Ames test (data not shown). We did not identify any mutation in *lasR* within a day of incubation when silencing of *mvfR* was observed, suggesting that it's silencing is not due to the loss of *lasR*. Nevertheless, the ability of 2-AA to promote *lasR* mutations was particularly pronounced after 10 d of incubation in isogenic *mutS* cells (Figure 5A), which are defective in DNA repair function. In contrast to the 2-AA dependent non-linear accumulation of *lasR* mutants observed in the WT background, a more linear accumulation of *lasR* mutants in the *mutS* mutant background is observed (Figure 5A). These data suggest that once a DNA repair mechanism is compromised, as in *mutS* mutants commonly found in *P. aeruginosa* cultures from CF sputum [37]–[38], 2-AA effect is more prominent.

**Table 1:** List of clones bearing *lasR, mexT*, and *fleQ* mutations.

Number of clones	Nucleotide position in *lasR*	Sequence change	Amino acid change
1	T452	Δ	Frameshift
2	C192G	C–G	Stop
1	C174G	C–G	Ala-Gly
2^*(2)^	T215	Δ	Frameshift
1	C692T	C–T	Ala-Val
1	C471G	C–G	Stop
4	G661T	G–T	Val-Leu
1	T235C	T–C	Cys-Arg
2^*(2)^	55–59	Δ	Frameshift
11^*(2) $(1)^	257–320	Δ	Δ62
2	67–573	Δ	Δ168
17	Full gene	Δ	Δfull

Mutations in the *mexT* and *fleQ* intergenic regions are indicated by * and $, respectively. The number in parentheses shows the number of clones bearing the respective mutation.

Although the mechanisms and advantages of *lasR* mutations are not clearly understood, it has been proposed that *lasR* mutation may provide a growth advantage [33]. We further tested the effects of 2-AA on long-term survival of *P. aeruginosa*. When grown under the condition of limited iron and aeration, PA14 cells reach stationary phase within 10–12 h and undergo significant lysis after 40 h (Figure 5B). However, addition of 2-AA inhibited entry into the lytic phase and the treated cells remained in stationary phase, promoting bacterial cell long-term survival (Figure 5B). To investigate whether 2-AA can also promote long-term survival *in vivo*, we developed a non-vertebrate, whole animal persistence infection assay in *Drosophila melanogaster*. Using the fly-*P. aeruginosa* feeding assay [28], flies were fed for 2 d with *P. aeruginosa* WT (PA14), 2-AA non-producer PA14 isogenic mutant strain *pqsA,* and the 2-AA producer strain *pqsB::C*. Flies were then transferred onto bacteria-free food, and the CFUs were quantified at 7 d post-feeding. Bacterial load decreased over time in the *pqsA-*infected flies, while flies infected with the 2-AA producing strains PA14 and *pqsB::C* sustained ∼3 log greater CFUs (Figure 5C), indicating that 2-AA helps bacteria to survive and persist, and thus promotes better fitness in a dynamic host environment. Due to the longer incubation duration, we cannot rule out the possibility that the persistence seen here may be due to *lasR* mutation induced by 2-AA. Together with the above *in vitro* studies, these *in vivo* results corroborate the view that 2-AA plays a key role in switching cells to a phase that promotes concomitant adaptations that enable *P. aeruginosa* to persist in a chronic infection.

### Synthesis of HAQs Analogs of Burkholderia are also Inhibited by 2-AA

Production of the 2-AA has been reported in plants, invertebrate animals, and vertebrate animals, including humans [41]. Moreover, several plant and human eubacterial pathogens, including *Pseudomonas*, *Bordetella*, *Burkholderia*, *Ralstonia*, *Streptomyces*, and *Mycobacteria*, as well as the Archeae *Sulfolobus*, encode putative PqsA and MvfR homologues, and in some cases, *pqsBCD*-related loci [42]. As such, these species might also produce 2-AA and use this molecule to down-regulate their respective virulence functions. Indeed, *Burkholderia thailandensis*, highly related and often used as a non-pathogenic surrogate for the level 3 pathogen *B. pseudomallei*, was confirmed to produce 2-AA (Figure S1). We assessed whether this 2-AA could inhibit HAQ biosynthesis in *B. thailandensis,* and found that exogenous 2-AA inhibited production of the HHQ and HNQ methylated analogs HMHQ and HMNQ (Figure S5A and B) [23], suggesting that 2-AA may perform analogous functions in *B. thailandensis* as observed in *P. aeruginosa.* 2-AA producing species may have a selective advantage in mixed microbial communities by adversely influencing the expression or activities of fitness traits of their neighbors.

Here we provide evidence that 2-AA, a small volatile molecule produced by *P. aeruginosa* (and *B. thailandensis*), is a QS regulated molecule and an important modulator of acute and chronic virulence functions. Although much work has focused on how QS cell-cell signaling leads to the activation of a plethora of virulence factors, little is known about the silencing of these factors. Degradation of AHL by an AHL acylase [43] and interference of LasR and RhlR binding to their promoter by the transcription factors QscR [44] and RsaL [45] have been shown to down-regulate AHL mediated QS. We recently demonstrated the existence of an interplay between QS systems, their components, and environmental factors that negatively impact HAQs [27]. However, no signaling molecule has been identified to date that negatively regulates acute virulence QS related functions or promotes bacterial adaptation and long-term persistence. Furthermore, no volatile compounds have been reported to be involved in QS regulation and virulence. Our results demonstrate that the low molecular weight molecule 2-AA silences the MvfR regulon via a negative feedback mechanism, most likely via repressed synthesis of MvfR ligands transcriptionally and post-transcriptionally, which restricts HAQ-mediated QS signaling and thus acute virulence functions, thereby enabling chronic infection. We propose the idea that there may be QS regulated aerial communication among bacteria, as 2-AA is a QS-regulated molecule and a volatile signal [46]. Such aerial communication may occur and be critical in a microenvironment such as the one found in biofilms and in infected wounds or CF lungs. It is important to note that suboptimal inhibitory concentration of 2-AA affects only a subpopulation of cells, suggesting that MvfR active and inactive cells co-exist causing a bistable phenotype. Similar bi/multistability phenotypes have been described as naturally occurring for several well known systems regulated by feedback mechanism, such as regulation of the Lac operon in *E. coli*, mucoidy in *P. aeruginosa*, lysogeny of the lambda phage, and transformation competency and sporulation in *B. subtilis* [24], [47]. In our model (Figure 5D), we propose that 2-AA inhibits MvfR via feedback regulation and promotes longer cell survival *in vitro* and *in vivo* in flies, presumably by interfering with cell lysis and inducing mutations in *lasR*. Additionally, the accumulation of *lasR* mutations that allow adaptation to low oxygen conditions [48] suggest that this mechanism contributes to overall pathogen fitness in chronic infections. Whether, *lasR* mutations arise in WT cells, MvfR silenced cells, and/or metabolically altered cells, remains to be determined. In corroboration, the *Drosophila* persistence assay showed that 2-AA promotes bacterial long-term survival and as such fitness.

Assessment of the potential role of 2-AA in the establishment and persistence of chronic infections has been hindered by a lack of clinically relevant chronic infection models. Nevertheless, the *Drosophila* persistence assay, using 2-AA producing and non-producing isogenic strains, permits us to at least determine the impact of this small molecule in a dynamic host environment that shares many immune functions with the human. Our identification of a *P. aeruginosa* QS regulated volatile molecule that limits virulence and invasive functions associated with acute infections while promoting phenotypic and genetic changes associated with chronic infection elucidates novel avenues for combating bacterial adaptation and survival in the chronic infection environment. These results also suggest that interfering with the MvfR pathway could prevent both acute and chronic infections and that 2-AA synthesis provides a potential target for the development of new anti-virulence drugs in the combatance of *P. aeruginosa*, and possibly of other pathogenic bacteria such as *Burkholderia.* This study uncovers insights that paradigmatically pave the way for the search for 2-AA-like volatile small molecules that promote pathogen adaptation and establishment of chronic infections caused by foreboding human pathogens.

## Materials and Methods

### Ethics Statement

This study was carried out in strict accordance with the recommendations in the Guide for the Care and Use of Laboratory Animals of the National Institutes of Health. The protocol was approved by the Committee on the Ethics of Animal Experiments of the Massachusetts General Hospital (Permit Number: 2006N000093/2). All Procedures were performed under sodium pentobarbital anesthesia, and all efforts were made to minimize suffering.

### Bacterial Strains and Growth Conditions

A *P. aeruginosa* strain known as Rif^R^ human clinical isolate UCBPP-PA14 (PA14) was used in the present experiments [11]. All of the PA14 mutants described in this paper are isogenic to UCBPP-PA14. The bacteria were grown at 37°C on LB broth or on plates of LB agar containing appropriate antibiotics unless otherwise indicated. The overnight PA14 cultures were grown in LB and diluted the following day in fresh media with or without 2-AA. Bacterial growth kinetics was determined by taking OD_600 nm_ measurements.

### LC/MS

Quantification of 2-AA and HAQs in bacterial culture supernatants was performed as described previously [14], [49]. The HAQs were separated on a C18 reverse-phase column connected to a triple quadrupole mass spectrometer, using a water/acetonitrile gradient [49]. Positive electrospray in MRM mode with 2×10^−3^ mTorr argon and 30 V as the collision gas and energy was employed to quantify 2-AA and HAQs, using the following ion transitions: 2-AA 136>91, HHQ 244>159, HHQ-D4 248>163, PQS 260>175, and PQS-D4 264>179. To quantify HAQs, Pseudomonas PA14 cells were grown in LB supplemented with different concentrations of 2-AA (Sigma, USA), with untreated LB being used as a negative control. The culture supernatant was collected at different stages of growth mixed with an equal volume of methanol, and the compounds were analyzed by LC/MS. All samples were analyzed in triplicate.

### Bacterial Growth Curves

Bacterial cells were diluted 1/100 from overnight cultures and grown in six replicates in 20 µl in a 96-well plate (Corning, Inc., Corning, NY). Bacterial growth was measured as a function of optical density at OD_600 nm_ using Tecan F200 automated plate reader (Infinite F200, Tecan Group Ltd, Männedorf, Switzerland).

For the *pqsA-sucB* assay, the bacteria were diluted from overnight cultures to OD_600 nm_ 0.1 in NaCl-free LB with tetracycline (50 µg/ml) and grown (Sigma, USA), with or without 10% sucrose (200 µl). Growth was measured in triplicate every 30 min for up to 25 h, using a Sunrise plate reader (Tecan Group Ltd, Männedorf, Switzerland).

For lysis experiments, the LB medium was treated with Chelex 100 resin 100–200 mesh, sodium glutamate (50 g/l)(BioRad, Hercules, CA) for 1 h followed by filtration with 0.2 µm filters (Corning, NY). Bacterial cells were diluted 1/100 in presence of various concentrations of 2AA. Triplicates (200 µl volumes) were inoculated in 96 wells plates. The plates were incubated and growth was recorded every 30 min for 3 d using a Sunrise plate reader (Tecan Group Ltd, Männedorf, Switzerland).

### *pqsA-*GFP(ASV) Fluorescence Measurement and *pqsA-*LacZ, ß-galactosidase Assay

PA14 or *pqsA::pqsH* cells carrying pAC37 plasmids with *pqsA* promoter fused to short-lived GFP {*pqsA-*GFP(ASV)} [50] were grown overnight in LB supplemented with gentamycin (50 µg/ml). The cultures were diluted in the morning with the test compound at desired concentrations and aliquoted (200 µl) into 6 replicates in a 96-well assay plate (Corning, Inc. Corning, NY). Green fluorescent protein (GFP) fluorescence (excitation at 485 nm, emission at 535 nm) and OD_600 nm_ for growth was measured every 30 min using a Tecan F200 automated plate reader (Infinite F200, Tecan Group Ltd, Männedorf, Switzerland). Values presented were above the background fluorescence from the empty strain. For measurement of ß-galactosidase activity, *pqsA::pqsH* cells harboring pGX5, which carries the *pqsA*-*lacZ* reporter gene [51], were diluted to OD_600 nm_ = 0.05; and OD_600 nm_ and ß-galactosidase activity (expressed as Miller Units) were measured at the indicated optical densities. Assays were performed in triplicate. All of the experiments were repeated at least three times.

### Construction of *pqsB::C*

The mutant was constructed as described by Lesic et al [52] such that most of the *pqsB* and *pqsC* genes were replaced with a Kanamycin resistance marker. The deletion was made from nt 151 in *pqsB* to nt 456 in *pqsC* (within the coding regions), leaving 150 bp of the 5′ end of the *pqsB* coding region and 149 bp of the 3′ end of the *pqsC* coding region. The mutants were selected on LB plates containing Kanamycin (200 µg/ml).

### Visualization of 2-AA Affected Cells

PA14 cells harboring the plasmid pAC37, which contained the *pqsA-*GFP(ASV) reporter fusion, were grown to mid-logarithmic phase in the presence of various concentrations of 2-AA. The cells were washed in phosphate buffered saline (PBS) and their membranes were stained with FM-64 (Invitrogen) according to the manufacturer's instructions. Five-microliter aliquots of bacterial cells were spotted onto slides and covered with Poly-L-lysine coated coverslips (Sigma Aldrich, US). The bacteria were visualized using an Eclipse E800 (Nikon) microscope with FM-64-stained membranes visualized as red and *pqsA-GFP* visualized as green. The pictures were processed using Spot V4.0.9 (Diagnostic Instruments) software.

### Plasmid Construction

To constitutively express *pqsABCD,* a genomic fragment containing *pqsABCD* was amplified by PCR using *pqsABamHI* 5′ CATGGATCCAACGTTCTGTCATGTCCACG3′ and *PqsDPst1* 5′CGACTGCAGTCAACATGGCCGGTTCAC3′ primers from an H44 cosmid [53] as template. The BamH1 and Pst1 digested PCR product was ligated to a pDN18 vector digested with BamH1 and Pst1. The construct was introduced into *E. coli* Top10 (Invitrogen) cells and *P. aeruginosa mvfR* mutant cells by electroporation.

To construct *pqsA-sucB* reporter fusion, the *pqsA* promoter was amplified using the primers 5′GACTAGTCGAGCAAGGGTTGTAACGGTTTTTG3′ and 5′GAAGATCTGACAGAACGTTCCCTCTTCAGCGA3′ and the PA14 chromosome was used as template. The *sacB* gene was amplified using the primer pairs 5′GAAGATCTATGAACATCAAAAAGTTTGCA3′ and 5′AAACTGCAGGTTGATAAGAAATAAAAGAAAATGCC3′ from pKOBEG-sacB plasmid [54]. PqsA promoter (PpqsA) was then digested with SpeI/BglII and the fragment containing sacB was digested with BglII/PstI. The two fragments were ligated to the CTX (TetR) plasmid digested with SpeI/BglII. The ligated vector was eletroporated into *E. coli* SM10 lambda pir and used to integrate the CTX-PpqsA-sacB to PA14 chromosome. Rif and TetR plates were used to select for the PA14 CTX-P*pqsA*-sacB clones and the presence of CTX-*PpqsA-sacB* in the PA14 chromosome was further confirmed by PCR.

### Pyocyanin Quantification

Overnight PA14 cultures were diluted to OD_600 nm_ = 0.05 in 5 ml LB or LB +200 µg/ml 2-AA. The bacteria were grown in triplicate at OD 3.0. Pyocyanin was extracted with chloroform from 5 ml cell culture supernatant and then extracted with an equal volume of HCl (0.2 N); optical density was measured at OD_520 nm_. The amount of pyocyanin was quantified by multiplying the OD_520 nm_ value by 17.072 to obtain values in µg/ml [55].

### Pyoverdine Quantification

PA14 bacterial cells (200 µl) were grown in 96-well plates in D-TSB medium with a range of 2-AA concentrations. The plates were incubated and pyoverdine production and growth were measured every 30 min by a Tecan F200 automated plate reader (Infinite F200, Tecan Group Ltd, Männedorf, Switzerland). Pyoverdine levels were measured using excitation at 400 nm and emission at 460 nm. The values were normalized to cell growth (OD_600 nm_). Pyoverdine concentrations were calculated using a calibration curve of fluorescence for a range of pyoverdine concentrations (Sigma Aldrich, USA).

### Mortality Studies

#### Fly mortality

Fly maintenance and the infection assays were performed as described previously [29]. For the survival assays, 50–60 flies were infected with a bacterial suspension of 5×10^7^ cells/ml (grown in LB to OD_600 nm_ = 3.0) in the presence (1.6 ng of 2-AA /fly) and absence of 2AA. The bacteria were mixed with 2-AA immediately before injection, and the flies were incubated at 21°C following infection. Fly mortality was assessed over time as described previously [29]. The experiment was performed twice with qualitatively similar results between the replicates. Bacterial inoculations were accomplished via pricking the middle dorsolateral thorax of the fly with a needle previously dipped into the bacterial suspension. The number of inoculated cells was ∼100 (2 nl) on average per fly. Statistical significance of survival kinetics was assessed by the Kaplan Meier method.

#### Mouse mortality

A thermal injury mouse model [32] was used, as described previously [11], to assess bacterial pathogenicity in 6-week-old CD1 mice (Charles River, Boston, MA, USA). The animal protocol was approved by the Massachusetts General Hospital Institutional Animal Care and Use Committee. Following administration of anesthesia, a full-thickness thermal burn injury involving 5–8% of the total body surface area was produced on the dermis of the shaved mouse abdomen, and an inoculum of 2.5×10^5^ PA14 cells in 100 µl of saline or 2-AA solution (67 µg of 2-AA in 100 µl saline/mouse, mixture prepared immediately before injection) was injected intradermaly into the burn eschar. Mortality was recorded for 1 week. CFU counts were performed for 5–6 mice per group. Local muscle biopsies underneath the eschar, and from adjacent muscle on either side of the burn eschar, 20 h post-burn and infection were collected and homogenized in 1 ml of 1× PBS. The samples were diluted and plated on LB-agar plates containing rifampicin (50 mg/L). The systemic spread of the bacteria was assessed by counting the CFUs in the blood at 20 h post-burn and infection.

### Fly Feeding Infection and Bacterial Persistence

Fly feeding on a *P. aeruginosa*-containing solution was performed as described previously [28]. Briefly, 5–7-day-old female Oregon-R flies (N = 26 per group) were fed for 1 d with a mixture of 0.05 ml of LB bacterial culture at OD_600 nm_ = 1.8, 1 ml of 20% sucrose, and 4 ml of water. Thus, the feeding mix contained a final concentration of 1% LB, ∼3×10^7^ bacteria/ml, and 4% sucrose. A sterile cotton ball was placed at the bottom of each fly vial and was impregnated with 5 ml of the feeding mix. The flies in each treatment group were sub-divided into three fly vials (13 flies per vial), sealed with a clean cotton ball, and incubated at 25°C. A day later, the flies were transferred to 50 ml plastic screw-cap tubes (10 flies per tube). The tubes were perforated with a heated 0.9-mm needle to enable aeration, while the caps were perforated with a heated 1.2-mm needle and covered with a 2.3 cm Whatman disc (Fisher scientific, USA). A 0.2-ml volume of 4% sucrose solution was dropped onto the Whatman disc and covered with parafilm to provide food for the flies with minimal contamination. New fly tubes were prepared daily and the flies were incubated at 25°C. CFU counts per fly were measured at the indicated time points by dipping 6–9 flies per time point in 95% ethanol for 3 s and letting them dry out on paper tissues (Kimwipes). Then each fly was ground up using a 1.5-ml tube pestle in 0.1 ml of LB, and 1:10 dilutions were plated on LB plates. Statistical analysis of the CFU data was performed using the Student's t-test.

### Assessment of Mutations

PA14 and *mutS* mutant were grown overnight in LB. The saturated culture was diluted 1:10 in triplicate in 4% sucrose with varying concentrations of 2-AA, or without 2-AA. The diluted cultures were incubated statically at 25°C for 10 d. The cells were then diluted and plated to allow the numbers of total colonies to be counted the following day. After 2 d of plating, the *lasR* cells were identified by their excessive pyocyanin production. The percentage of *lasR* mutants in each sample was calculated and statistical significance was determined by Student's t-test, assuming equal variance. The *lasR* mutant colonies were further confirmed by sequencing of the complete *lasR* gene. In addition, the complete sequences of *mexA, mexE, mexS, mexT, mexZ, wspF, fleQ, exsA, accC, vfR, popD, mutS, nfxB, rpoN, rhlR, pqsA, pqsb, pqsE, pqsH, and mvfR* were also determined in these *lasR* mutant colonies.

## Supporting Information

Figure S1 **Production Kinetics of 2-AA in** ***Burkholderia thailandensis.*** Production kinetics of 2-AA at different stages of growth. Growth is shown as OD 600 nm. The error bars represent the standard deviation from 3 replicates.
(EPS)

Figure S2 **Levels of** ***2-AA in various isogenic mutants of PA14.*** 2-AA levels were quantified by LC/MS and presented as relative to WT (PA14) levels at OD 4.0.
(EPS)

Figure S3 **DHQ does not affect the expression of the** ***pqsA*** **promoter, neither 2-AA nor DHQ inhibit bacterial growth.** (A) Expression of *pqsA-GFP*(ASV) in the presence of increasing concentrations of DHQ measured as GFP fluorescence and expressed as relative fluorescence over time. (B and C). Growth curve of PA14 cells carrying the *pqsA-GFP*(ASV). Growth is represented as the OD 600 nm with increasing concentration of DHQ (B) and 2-AA (C) respectively. The values presented are average from 6 replicates.
(EPS)

Figure S4 **Growth Kinetics of PA14 and** ***pqsB::C*** **mutant carrying** ***pqsA-SacB*** **reporter**. Growth (OD 600 nm) was recorded in the presence or absence of sucrose (10%) and/or with exogenously added various concentrations of 2-AA. The *pqsA* promoter of *pqsA-SacB* in the *pqsBC* mutant was induced by exogenous addition of 10 µg/ml HHQ where indicated. The vertical line represents the growth at 20 hours post inoculation.
(EPS)

Figure S5 **2-AA inhibits the HAQ analogs in** ***B. thailandensi.*** Production kinetics of HMHQ (**A**) and HMHNQ (**B**) in *B. thailandensis* supernatants at different growth stages, minus and plus 2-AA. LC/MS data are averaged for triplicate samples.
(EPS)
