# Correction: A Quorum Sensing Regulated Small Volatile Molecule Reduces Acute Virulence and Promotes Chronic Infection Phenotypes

**DOI:** 10.1371/annotation/a1b0d6de-c6b4-4a5f-97ac-d500fbde806f

**Published:** 2011-08-23

**Authors:** Meenu Kesarwani, Ronen Hazan, Jianxin He, YokAi Que, Yiorgos Apidianakis, Biliana Lesic, Gaoping Xiao, Valérie Dekimpe, Sylvain Milot, Eric Deziel, François Lépine, Laurence G. Rahme

The fourth author's name was spelled incorrectly. The correct name is: Yok-Ai Que. The correct citation is: Kesarwani M, Hazan R, He J, Que YA, Apidianakis Y, et al. (2011) A Quorum Sensing Regulated Small Volatile Molecule Reduces Acute Virulence and Promotes Chronic Infection Phenotypes. PLoS Pathog 7(8): e1002192.doi:10.1371/journal.ppat.1002192

